# The lipase cofactor CGI58 controls placental lipolysis

**DOI:** 10.1172/jci.insight.168717

**Published:** 2023-05-22

**Authors:** Jennifer Guerrero-Santoro, Mayumi Morizane, Soo-Young Oh, Takuya Mishima, Julie P. Goff, Ibrahim Bildirici, Elena Sadovsky, Yingshi Ouyang, Vladimir A. Tyurin, Yulia Y. Tyurina, Valerian E. Kagan, Yoel Sadovsky

**Affiliations:** 1 Magee-Womens Research Institute, Department of Obstetrics, Gynecology and Reproductive Sciences, University of Pittsburgh, Pittsburgh, Pennsylvania, USA.; 2 Department of Obstetrics and Gynecology, Washington University School of Medicine in St. Louis, St. Louis, Missouri, USA.; 3 Center for Free Radical and Antioxidant Health, Department of Environmental and Occupational Health;; 4 Department of Chemistry;; 5 Department of Pharmacology and Chemical Biology;; 6 Department of Radiation Oncology; and; 7 Department of Microbiology and Molecular Genetics, University of Pittsburgh, Pittsburgh, Pennsylvania, USA.

**Keywords:** Reproductive Biology, Embryonic development

## Abstract

In eutherians, the placenta plays a critical role in the uptake, storage, and metabolism of lipids. These processes govern the availability of fatty acids to the developing fetus, where inadequate supply has been associated with substandard fetal growth. Whereas lipid droplets are essential for the storage of neutral lipids in the placenta and many other tissues, the processes that regulate placental lipid droplet lipolysis remain largely unknown. To assess the role of triglyceride lipases and their cofactors in determining placental lipid droplet and lipid accumulation, we assessed the role of patatin like phospholipase domain containing 2 (PNPLA2) and comparative gene identification-58 (CGI58) in lipid droplet dynamics in the human and mouse placenta. While both proteins are expressed in the placenta, the absence of CGI58, not PNPLA2, markedly increased placental lipid and lipid droplet accumulation. These changes were reversed upon restoration of CGI58 levels selectively in the CGI58-deficient mouse placenta. Using co-immunoprecipitation, we found that, in addition to PNPLA2, PNPLA9 interacts with CGI58. PNPLA9 was dispensable for lipolysis in the mouse placenta yet contributed to lipolysis in human placental trophoblasts. Our findings establish a crucial role for CGI58 in placental lipid droplet dynamics and, by extension, in nutrient supply to the developing fetus.

## Introduction

The placenta plays a vital role in supporting the growth and development of the eutherian fetus. Placental nutritional supply to the fetus includes triglycerides and other lipids, which account for more than half of the fetal caloric accretion during the second half of human pregnancy (1–8). Specifically, long-chain polyunsaturated fatty acids (LCPUFAs) are necessary for fetal brain and eye development. Indeed, unlike the trafficking of most fatty acids, which reflects their concentration in the maternal plasma (8), there is preferential transplacental transfer of the LCPUFAs docosahexaenoic acid (DHA) and arachidonic acid (AA) when compared with short-chain fatty acids, resulting in their higher concentration in the fetal blood than in the maternal blood (1, 9–15). Because the overall feto-placental lipogenic capacity does not meet intrauterine growth needs, maternal lipid supply is indispensable. Not surprisingly, pregnancy is characterized by increased maternal gut lipid absorption, hyperlipidemia, and augmented maternal adipose stores (16).

Maternal blood triglycerides (TGs) within chylomicrons or very low-density lipoproteins must be hydrolyzed by lipoprotein lipases to fatty acids and glycerol prior to their uptake by placental trophoblasts (14). Fatty acids enter trophoblasts by passive diffusion or through enhanced transport by specific fatty acid transporters (17, 18). In the cytosol, lipids bind fatty acid binding proteins for trafficking to intracellular compartments (19), including lipid droplets (LDs), where fatty acids are re-esterified for efficient storage in LDs and for prevention of cell injury by free fatty acids (20, 21).

Serving as an intracellular lipid depot, LDs comprise a core of TGs and cholesterol, within a monolayer of phospholipids, with attached regulatory proteins (22, 23). LDs are highly dynamic structures, modulated by a balance of uptake, storage, transport, and metabolism (24). The formation of LDs is an intricate process that requires enzymes and cofactors (25). LD hydrolysis is an intricate process, requiring the action of adipose triglyceride lipase (ATGL, PNPLA2, desnutrin) (26–28), which belongs to a family of patatin-like phospholipases (PNPLA1–9) with diverse lipolytic functions (29). PNPLA2 is expressed in white and brown adipose tissue and in many other organs. PNPLA2-knockout (PNPLA2-KO) mice exhibit mild obesity and die from heart disease after 12 weeks, reflecting their inability to mobilize fatty acids for fuel (20, 30–32). PNPLA2 is regulated by G_0_/G_1_ switch gene (G0S2), cAMP, perilipins (PLINs), ER/Golgi, and endocytosis proteins (33).

LD breakdown requires the interaction of PNPLA2 with its cofactor, comparative gene identification-58 (CGI58, also known as α/β hydrolase domain 5, ABHD5) (34–36), which was identified by comparative genomics analysis between *C*. *elegans* and humans (37). CGI58 is critical for defining substrate specificity and hydrolysis of LD TGs (38–40). Although CGI58 lacks lipid hydrolase activity (38), it broadens the affinity of PNPLA2 to glycerol-bound fatty acids to include lipolysis at the sn-1 position, in addition to sn-2 (38, 41, 42).

In humans, function-perturbing mutations in CGI58 cause neutral lipid storage disease with ichthyosis (NLSDI, also known as Chanarin-Dorfman syndrome), a rare autosomal recessive and severe multiorgan TG accumulation disease (43–46), in which the skin phenotype reflects the absence of the acylceramide intermediates essential for skin cornification (47). Crossing of *Cgi58*-heterozygous (*Cgi58*-Het) mice leads to Mendelian distribution of their progeny, yet KO mice die within 16 hours after birth with a phenotype that resembles its human counterpart (48). In contrast, PNPLA2 deficiency in humans causes neutral lipid storage disease with myopathy, characterized by systemic TG accumulation and cardiac and skeletal myopathy, with less hepatomegaly and liver steatosis when compared with NLSDI (46, 49).

In the absence of lipolysis, CGI58 is tethered through its tryptophan-rich N-terminal domain to LD surface PLIN proteins and LD phospholipids (50), rendering PNPLA2, at the LD surface, inactive (26, 42, 51–54). The phosphorylation of PLIN, dissociation of CGI58 from PLIN proteins, and interaction of CGI58 with PNPLA2 are critical steps for activating PNPLA2 and subsequent lipolysis and regulated by PKA-dependent lipase phosphorylation and by PLIN ligands (38, 55–58). During lipolysis, PNPLA2 converts triacylglycerol (TAG) to diacylglycerol (DAG). In the second step, DAG is hydrolyzed by hormone-sensitive lipase (HSL) to monoacylglycerol (MAG), which is subsequently hydrolyzed by monoglyceride lipase (26, 36, 59–62).

A unique aspect of placental lipid mobilization to the fetus is the need for lipolysis not only for satisfying placental metabolic and energetic requirements but also to regulate the release of fatty acid to the developing fetus. While critically important, placental LD dynamics remain understudied. We have previously shown that the accumulation of TGs in trophoblastic LDs is enhanced by hypoxia and by activation of PPARγ/retinoic acid receptor pathways (63–65). We also showed the regulatory role of PLIN2 in this process (66). The regulation of LD lipolysis in trophoblasts has not been hitherto studied to our knowledge. Here, we hypothesized that PNPLA2 and its cofactor CGI58 regulate lipolysis at trophoblastic LDs. We show that CGI58 is essential for mouse and human placental LD lipolysis. While PNPLA2 is critical for lipid accumulation in many organs, its function in the placenta appears dispensable. Using CGI58 protein as bait, we identified PNPLA9 as a CGI58-interacting protein and showed the function of PNPLA9 in TG accumulation in placental LDs.

## Results

### The lipase cofactor CGI58 plays a central role in trophoblastic LD accumulation.

**Figure 1:**
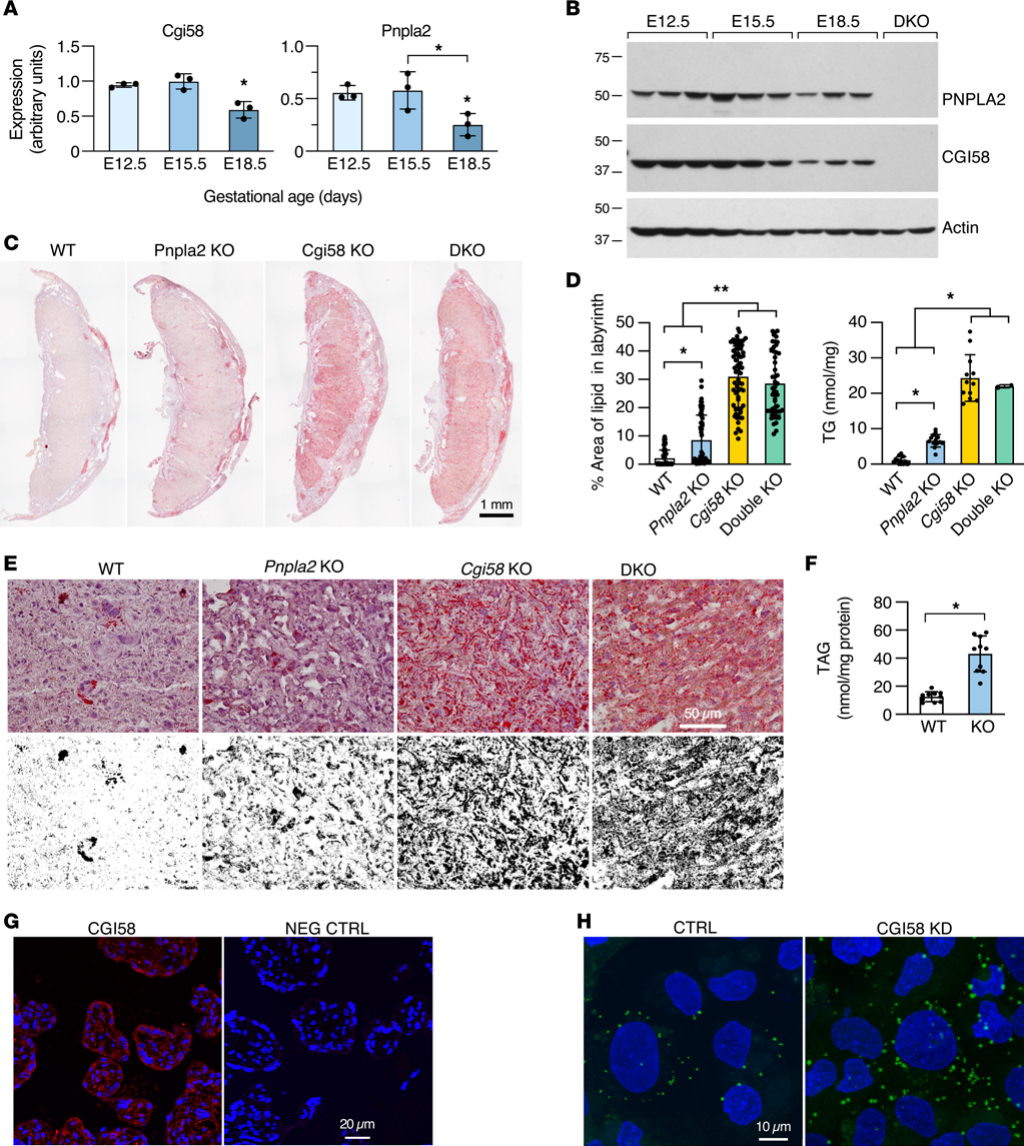
The function of Pnpla2 and Cgi58 in the placenta. (**A** and **B**) The expression of *Pnpla2* and *Cgi58* RNA (**A**): *n* = 3, *P < 0.05, ANOVA with post hoc Tukey’s test; and protein (**B**) across the second half of mouse pregnancy (*n* = 5). DKO, double KO. (**C**) The effect of ablation of *Atgl*, *Cgi58*, or both on placental lipid accumulation at E17.5, monitored using Oil Red O staining for neutral lipids, performed as detailed in Methods. WT, wild-type. (**D**) The accumulation of LDs in the E17.5 mouse placental labyrinth (left, *n* = 48–63) or TGs (right, *n* = 3–18) according to mouse genotypes. *P < 0.01, **P < 0.001, ANOVA with post hoc Tukey’s test. (**E**) The accumulation of LDs in the E17.5 mouse placental labyrinth, analyzed using bright-field microscopy enhancement as descried in Methods. (**F**) The level of TAG in the mouse placenta in WT and *Cgi58*-KO placentas, analyzed by MS/MS (*n* = 10, *P < 0.0001, *t* test). (**G**) Immunofluorescence staining of CGI58 (shown in red), with nuclei stained with 4′,6-diamidino-2-phenylindole (DAPI) (blue), in human placental villi. (**H**) Knockdown of *CGI58* in PHT cells, stained with boron dipyrromethene difluoride 493/503 (BODIPY) (green) for LDs and DAPI (blue) for nuclei.

We previously showed that exposure of primary human trophoblasts (PHT cells) to hypoxic injury led to accumulation of TGs within LDs. This process was PLIN2 dependent and driven by decreased oxidation and reduced fatty acid efflux (66). As expected, PHT cells also amassed LDs when exposed to diverse types of fatty acids, with a variable effect on cell phenotype (Supplemental Figure 1, A–D; supplemental material available online with this article; https://doi.org/10.1172/jci.insight.168717DS1). Considering the scant data on pathways regulating trophoblastic LD lipolysis, we sought to first assess the expression of proteins that might be involved in trophoblastic lipolytic pathways. PNPLA2 has been shown to be expressed in the placenta (67). Assessing the expression of HSL, PNPLA2, and the PNPLA2 cofactor CGI58, we detected the expression of transcripts for *CGI58* and *PNPLA2*, but not *HSL*, in PHT cells cultured in standard or hypoxic conditions when compared with other human tissues (Supplemental Figure 1E). *Pnpla2* and *Cgi58* were also expressed in the mouse placenta (Figure 1, A and B).

To analyze the role of PNPLA2 and its cofactor CGI58 in placental lipolysis, we examined placental LD accumulation in mice deficient for *Pnpla2*, *Cgi58*, or both (DKO, Figure 1C). Notably, it was previously reported that both *Pnpla2-* and *Cgi58*-KO mice were born at the expected Mendelian distribution, and while *Pnpla2*-KO mice survived to adulthood, *Cgi58*-KO newborns died 2 days after delivery from the phenotype described earlier. We verified these findings, noting small differences in fetal or placental weight in the *Pnpla2*-KO or *Cgi58/Pnpla2-*DKO mice, compared with controls (Supplemental Figure 2, A–C). Importantly, when compared with WT or *Cgi58*-Het mice, *Cgi58*-KO mice exhibited marked accumulation of neutral lipids in the placenta (Figure 1, C–E). *Pnpla2*-KO placentas showed a small, yet significant, neutral lipid accumulation, with no additional effect in *Cgi58/Pnpla2*-DKO mice compared to *Cgi58*-KO mice (Figure 1D). Tandem mass spectrometry (MS/MS) analysis of the *Cgi58*-KO placenta also verified elevated total TAG levels (Figure 1F), particularly in LCPUFA species containing 20:4, 22:4, 22:5, and 22:6 acyl chains (Supplemental Table 1).

As expected, CGI58 was also expressed in human villous trophoblasts. We validated the effect of CGI58 deficiency using knockdown (KD) of CGI58 in PHT cells, showing accumulation of LDs in these cells (Figure 1, G and H). To rule out the possibility that the effect of CGI58 ablation was mediated by altered expression of PLIN2, which promotes placental LD formation (66), we showed that KD of *CGI58* had no effect on PLIN2 or PLIN3 mRNA in PHT cells (Supplemental Figure 2D). Similarly, KD of *CGI58* had no effect on PNPLA2 expression in PHT cells (Supplemental Figure 2E).

**Figure 2:**
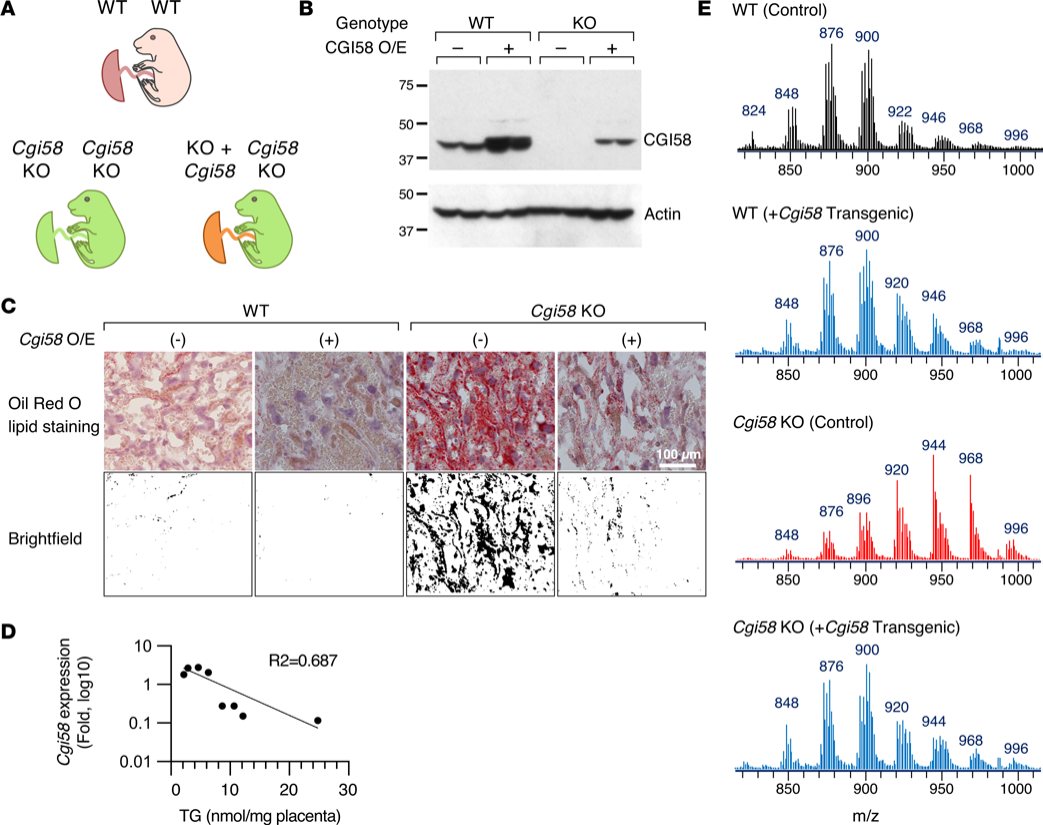
Rescue of the lipid accumulation phenotype in *Cgi58*-KO mice by placenta-selective overexpression of CGI58. (**A**) A schematic depicting our strategy for rescue of the *Cgi58*-mediated placental phenotype. (**B**) Western blot, showing overexpression of CGI58 in WT or *Cgi58-*KO placentas. (**C**) Oil Red O staining of the WT versus *Cgi58*-KO mouse placenta, analyzed using bright-field microscopy enhancement as descried in Methods. (**D**) The ratio of mouse *Cgi58* expression (based on variable CGI58 expression in several individual *Cgi58*-KO placentas) to placental TG levels, analyzed using R^2^-goodness-of-fit. (**E**) MS/MS analysis of TAG’s fatty acid distribution across the phenotypes as noted in the figure, expressed as *m/z* ratio.

To ensure that lipid accumulation in the *Cgi58*-KO mouse placenta indeed reflected the deletion of placental *Cgi58* and not an indirect effect by other fetal organs, we deployed lentivirus-driven overexpression of *Cgi58* selectively in the blastocyst, which leads to overexpression of the *Cgi58* transgene in the trophectoderm and, later, the placenta, but not in the fetus (Figure 2A) (68–70). Notably, we previously showed that this approach leads to transgene overexpression in the mouse placental labyrinthine and junctional zones (70). Because the mouse ICR (CD1) line was needed for this lentivirus-driven manipulation, we first validated that fetuses and placentas from transferred ICR blastocysts resembled those derived from C57BL/6 blastocysts (Supplemental Figure 3A). We verified that the lentiviral transduction rescued CGI58 expression in the *Cgi58*-KO placenta, with no effect on feto-placental weight (Figure 2B and Supplemental Figure 3B). Importantly, we found that the placenta-selective CGI58 rescue in the *Cgi58*-KO mouse markedly lowered placental lipid levels, and, outstandingly, restored the distribution of TAG-bound fatty acids in placental TAG (Figure 2, C–E). We also verified that the rescue effect was selective to the placenta and not observed in the fetal liver (Supplemental Figure 3C). Together, these data establish the potentially unique role of CGI58 in the accumulation of LDs and neutral lipids in the placenta.

### Placental PNPLA9 interacts with CGI58.

**Figure 3:**
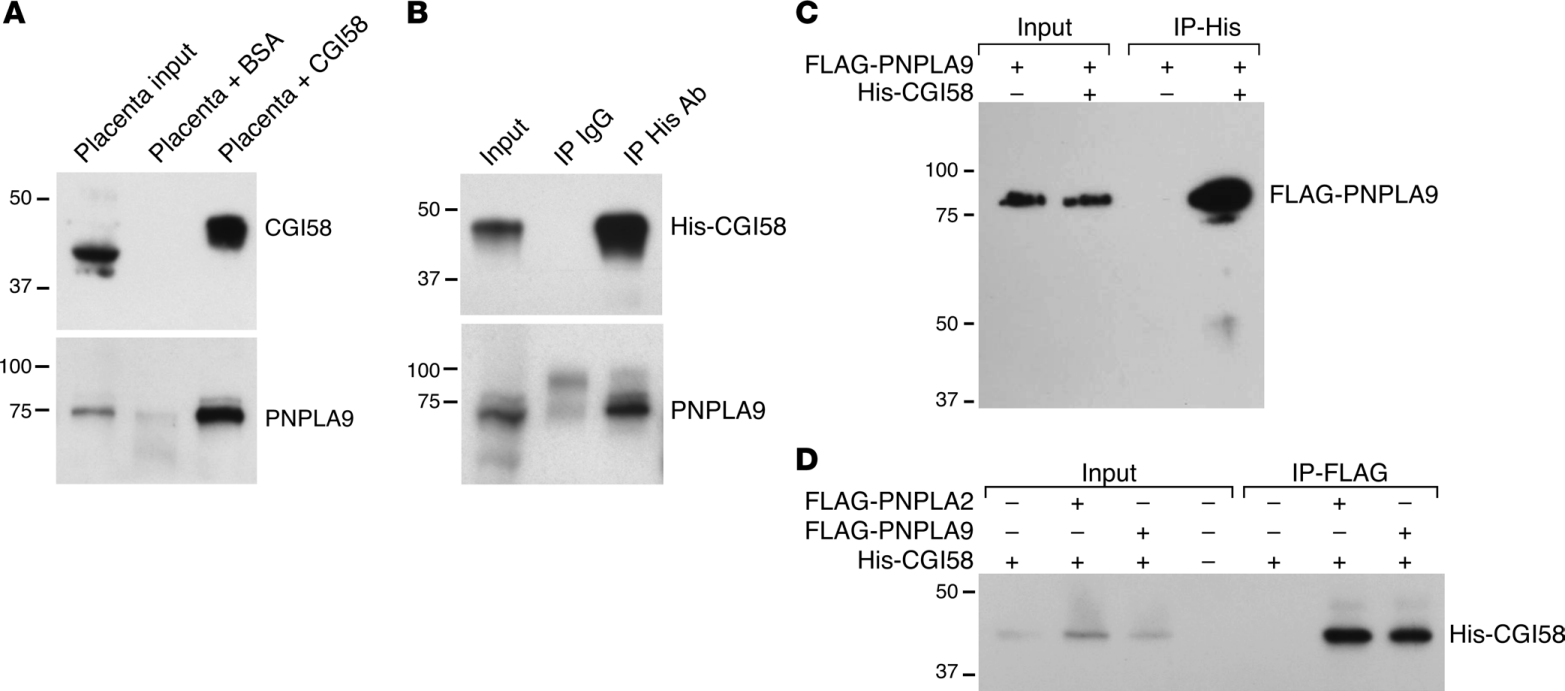
CGI58 interacts with PNPLA9 in the placenta. (**A**) CGI58 cross-linked with Sulfo-SBED interacts with endogenous Pnpla9 in mouse placenta. Purified BSA and His-CGI58 were UV–cross-linked with Sulfo-SBED and mixed with WT mouse placental lysate, then pulled down using streptavidin beads as detailed in Methods and processed for Western blot (WB) for CGI58 and PNPLA9. Note that CGI58 was conjugated to Sulfo-SBED in the third lane and, hence, shows a higher band. (**B**) Purified His-CGI58 interacts with endogenous PNPLA9 in a mouse placenta lysate. The immunoprecipitations were performed overnight with either IgG (control) or anti-His antibody and processed for His-CGI58 or PNPLA9 blots, as detailed in Methods. (**C**) Overexpressed His-CGI58 interacts with overexpressed FLAG-PNPLA9 in HEK293T cells. Transfections, overnight immunoprecipitation (IP), and blotting were performed as detailed in Methods. WB was performed using anti-FLAG antibody. (**D**) Overexpressed His-CGI58 interacts with overexpressed FLAG-PNPLA2 or FLAG-PNPLA9 in HEK293T cells. The experiment was performed as in panel **C**, with IP of FLAG and the addition of FLAG-PNPLA2. WB was performed using anti-His antibody. Note that all panels depict a representative experiment, each repeated at least 3 times.

Considering the relatively minor role of PNPLA2 in trophoblastic LD accumulation, we surmised that other lipases might interact with CGI58 and mediate LD lipolysis in trophoblasts. To identify additional lipases that might interact with CGI58, we used Sulfo-SBED to cross-link CGI58 with putative interacting proteins present in a placental tissue lysate. After UV irradiation, the cross-linked CGI58 and its potential partners were precipitated using streptavidin beads and resolved using SDS-PAGE gel. The gel slices were cut and analyzed by MS proteomics. Among the interacting peptides in the 65 to 80 kDa gel slice (Supplemental Table 2), we identified the phospholipase and triglyceride lipase PNPLA9 (PLA2G6) as a potential CGI58-interacting partner. We validated the interaction of PNPLA9 with CGI58 by pulling down the Sulfo-SBED–biotinylated CGI58 and showed that endogenous PNPLA9 indeed associated with CGI58 compared with BSA control (Figure 3A). We corroborated this interaction using purified His-tagged CGI58 as bait for PNPLA9 from a mouse placental lysate (Figure 3B). Consistently, by immunoprecipitating His-CGI58 in HEK293T cells coexpressing FLAG-PNPLA9 and His-CGI58, we found that FLAG-PNPLA9 associated with CGI58 compared with control cells expressing FLAG-PNPLA9 alone (Figure 3C). Reassuringly, we also found that His-CGI58 interacted with FLAG-PNPLA2 or FLAG-PNPLA9 once we pulled down the FLAG fusion proteins (Figure 3D).

### The function of PNPLA9 in trophoblastic LD accumulation.

We first assessed the expression of PNPLA9 in the human and mouse placenta. As shown in Supplemental Figure 4, A and B, PNPLA9 is expressed in human placental villi, mainly in the trophoblast layer, with scattered expression in the villous core, and across all layers of the mouse placenta. Using laser-capture microdissection, we quantified the relative expression of mouse *Pnpla9*, also validating a similar expression pattern for *Cgi58* and *Pnpla2* (Supplemental Figure 4C).

**Figure 4:**
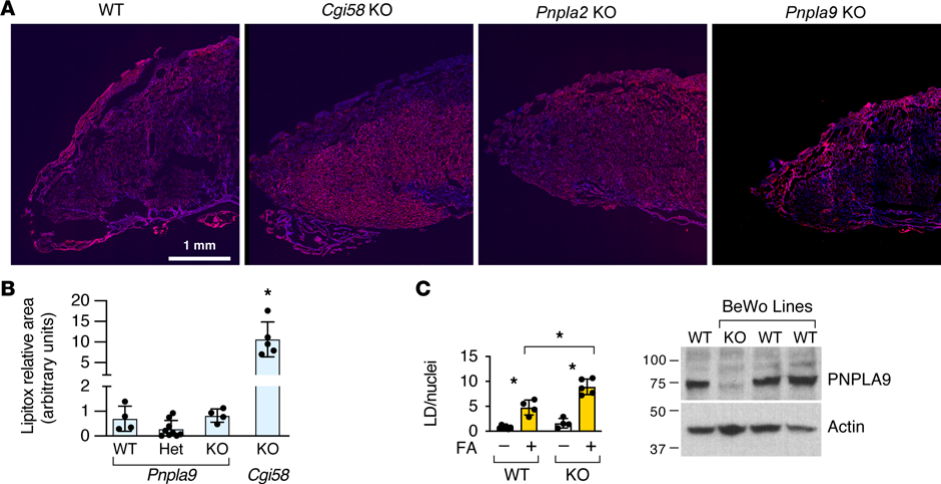
PNPLA9 plays a minor role in placental lipid accumulation. (**A**) LipidTox red fluorescence analysis of lipid accumulation in the placentas of different mouse phenotypes. Note the pattern of accumulation in the *Cgi58-*KO mouse. (**B**) Quantitation of lipid accumulation in *Pnpla9* mouse genotypes, using *Cgi58-*KO mice as controls (*n* = 4–9, *P < 0.001, ANOVA with Tukey’s post hoc test). (**C**) Accumulation of LDs in CRISPR/Cas9-generated *PNPLA9*-KO BeWo human trophoblast line in the absence or presence of added fatty acid mix for 48 hours, normalized to the number of nuclei and analyzed as described in Methods (*n* = 4–6, *P < 0.01, ANOVA with Tukey’s post hoc test). The Western immunoblotting validates PNPLA9-KO efficiency.

We used *Pnpla9*-KO mice to explore the potential role of PNPLA9 in placental lipid accumulation. We examined LD accumulation in WT, *Pnpla9*-Het, or *Pnpla9*-KO placentas, and as expected, we found no significant difference in fetal or placental weights among these genotypes (Supplemental Figure 4D). Importantly, we found no difference in LD accumulation in the *Pnpla9*-KO mouse placenta (Figure 4, A and B). We also used MS/MS to show that mouse KO of *Pnpla9* did not affect the distribution of TAG-bound placental fatty acids (compare Supplemental Figure 4E to Figure 2E). Moreover, crossing mice to produce *Pnpla2/Pnpla9* Het/Het, Het/KO, and KO/KO fetuses had an insignificant effect on placental TG accumulation (Supplemental Figure 4F). Interestingly, using CRISPR/Cas9 to knock out *PNPLA9* in a BeWo trophoblast line, we found no difference at baseline, as expected, but a significant increase in accumulation of LDs in *PNPLA9*-KO cells upon addition of a linoleic acid/oleic acid (LA/OA) fatty acid mix for 48 hours (Figure 4C), with an insignificant difference in LD accumulation between WT and *PNPLA9*-KO BeWo trophoblasts that were exposed to LCPUFAs (Supplemental Figure 4G). Together, these results establish the dominant role of CGI58 in placental lipid accumulation, with a minor role for PNPLA9 in LD dynamics in human trophoblasts.

### The expression and function of PNPLA2, PNPLA9, and CGI58 in hypoxic placentas.

**Figure 5:**
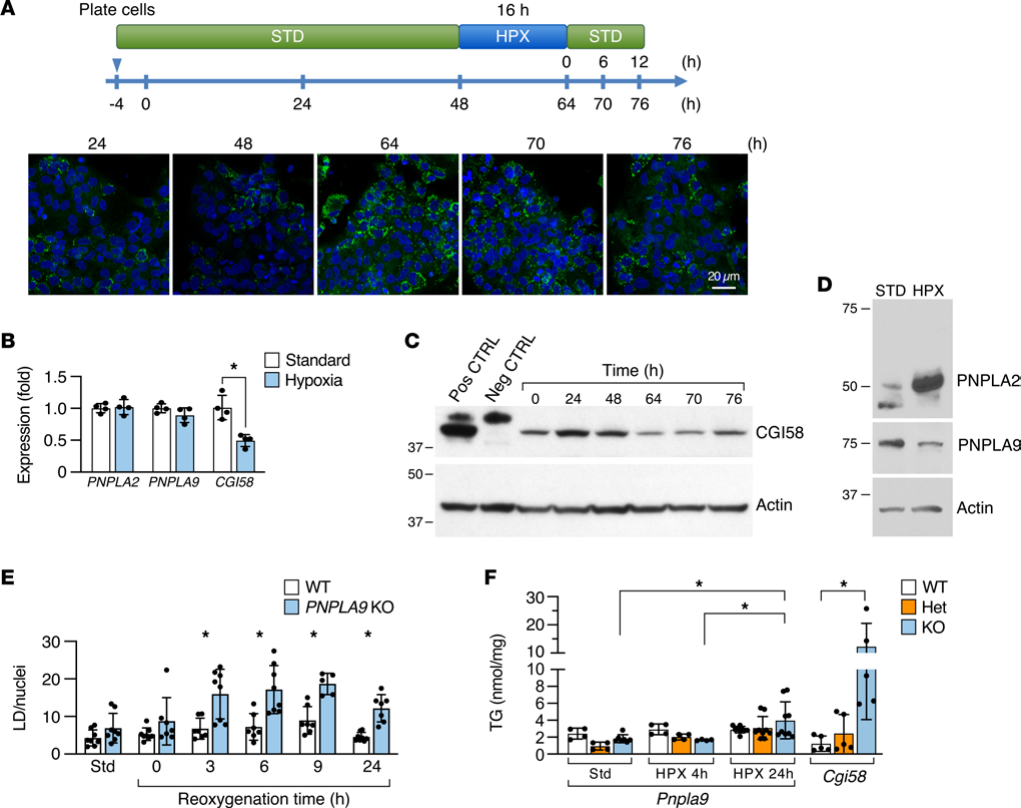
The function of CGI58 and PNPLA9 in the hypoxic placenta. (**A**) LD accumulation in trophoblasts during hypoxia and reoxygenation. PHT cells were exposed to standard culture conditions for 48 hours, then to hypoxia for 16 hours (48–64 hours), and then back to standard conditions for an additional 12 hours, as detailed in Methods and shown in the upper panel. The images show LD accumulation over the experimental time course. (**B**) The expression of *PNPLA2*, *PNPLA9*, and *CGI58* mRNA in hypoxic (48–72 hours) PHT cells (*n* = 4, *P < 0.05, paired *t* test). (**C**) The expression of CGI58 protein along the time course in **A**. (**D**) The expression of PNPLA protein along the time course in **A** (representative experiments, *n* = 3). (**E**) The effect of *PNPLA9* KO on LD accumulation in BeWo cells (*n* = 7–8, *P < 0.05, ANOVA with Tukey’s post hoc test). (**F**) The effect of hypoxia on LD accumulation in the E17.5 mouse placenta, based on Pnpla9 genotype, with *Cgi58*-KO mice, maintained in room air, used as a positive control (*n* = 4–8, *P < 0.05, ANOVA with Tukey’s post hoc test).

We previously showed that hypoxia increases lipid accumulation in trophoblasts by reducing β-oxidation and neutral lipid efflux but without affecting lipid uptake or TG synthesis (66). We therefore surmised that hypoxia might reduce the expression of CGI58 or its associated lipases and thus diminish the breakdown of neutral lipids from their LD reservoir. As expected, culturing PHT cells in hypoxia led to LD accumulation (Figure 5A and Supplemental Figure 5, A and B). Importantly, this was associated with reduced expression of CGI58 mRNA and protein, yet increased PNPLA2, and led to a small reduction in PNPLA9 protein levels (Figure 5, B–D, and Supplemental Figure 5C). We also showed that KO of *PNPLA9* in BeWo cells led to further accumulation of cellular LDs in cells exposed to hypoxia and reoxygenation (Figure 5E).

To determine whether hypoxia and reoxygenation of the mouse dam in vivo enhances lipid accumulation, we placed pregnant dams in hypoxia (O_2_ = 11%) between E12.5 and E17.5 and analyzed lipid accumulation after exposure to hypoxia. Notably, there was no difference in fetal or placental weight among the paradigms (Supplemental Figure 5D), with no further accumulation of placental LDs in hypoxic *Cgi58*-KO placentas (Supplemental Figure 5E). After reoxygenation of the *Pnpla9*-KO mice for 4–24 hours before sacrifice, we found a small increase in accumulation of placental TGs. This increase was strikingly lower than the accumulation of neutral lipids in the *Cgi58* mice on room air (Figure 5F). Together, these data support the role of CGI58 in regulating lipid efflux from placental LDs during hypoxia and suggest a more minor role for PNPLA9 in that context.

## Discussion

In our quest to understand lipid mobilization across the placenta, we centered our investigation on the LD depot in trophoblasts and on key lipases and their cofactors that regulate the initial lipolytic steps. Thus far, data on these processes in the context of placental biology are scarce, showing that the expression of CGI58 mRNA is increased by 2-fold in placentas from pregnancies complicated by gestational diabetes or maternal obesity, with no change in PNPLA2 in these conditions, and reduced by nearly 2-fold in placentas from women with preeclampsia (71–73). We found that CGI58 was expressed in human and mouse trophoblasts and that CGI58 was essential for placental lipid mobilization, with intense lipid accumulation in the placentas of CGI58-KO fetuses, which was corrected upon selective overexpression of CGI58 in the placenta. Notably, although CGI58 was expressed in the placental labyrinthine and junctional zones, fat accumulated mainly in the labyrinthine zone of the CGI58-KO mouse placenta, which plays a dominant role in maternal fetal oxygen and nutrient exchange. Although CGI58 is identified as a cofactor of PNPLA2 and required for the TAG hydrolase activity of PNPLA2 (38, 54), our results indicate that the role of PNPLA2 is minor. Our observations resemble the more widespread phenotype of human or murine CGI58 mutations, causing hepatomegaly, hepatosteatosis, and ichthyosis, when compared with the more restricted phenotype of PNPLA2 deficiency (46–48, 74).

The phenotypic differences between deficiency of CGI58 or PNPLA2 suggest that CGI58 may have other functions (75). Indeed, CGI58 is implicated in regulation of inflammation and insulin resistance (76) and lysophosphatidic acid–specific acyltransferase activity (77, 78). The relevance of these activities to placental function is currently unknown. An alternative explanation is that CGI58 may be a cofactor for the activity of other lipases (reviewed in ref. 79). To examine this possibility, we used a protein-protein interaction assay, using CGI58 as bait, and identified PNPLA9 as a CGI58-interacting protein in mouse placental tissues and in human trophoblasts. We validated this interaction using several protein-protein immunoprecipitation approaches, where WT or His-tagged CGI58 was used as bait to pull down PNPLA9 from mouse placental lysate, with immunoprecipitation validated in human cells. Finally, we highlight that other CGI58-interacting proteins that were identified in our protein-protein interaction assay, and other proteins that were expressed at a lower level and below our assay sensitivity, may be relevant to modulation of CGI58 action. Notably, PNPLA2 was not identified in our screening assay, yet we validated its interaction with CGI58 by immunoprecipitation.

The family of PNPLA proteins includes at least 10 proteins, with members exhibiting acyl hydrolase activity or phospholipase activity. Some members, including PNPLA2 and PNPLA9, include both functions (27; reviewed in refs. 29, 80, 81). We showed that PNPLA9 is expressed in human and mouse trophoblasts. Although PNPLA9 deficiency, alone or in combination with PNPLA2 deficiency, did not significantly affect lipid accumulation in the mouse placenta, we observed a more pronounced effect on LDs and neutral lipids in human placental cells, implying a role for PNPLA9 in human trophoblasts.

We previously showed that human placentas from pregnancies complicated by fetal growth restriction related to reduced placental perfusion exhibit lipid retention and that LD accumulation in hypoxic trophoblasts can be attributed to reduced lipid oxidation and efflux (66). Here, we examined the effect of hypoxia on the expression of placental PNPLA2, PNPLA9, and CGI58. We found that hypoxia led to reduced expression of CGI58 mRNA and protein, with a weak effect on the expression of PNPLA9, and increased expression of PNPLA2. Together, our data suggest that diminished expression of CGI58 contributes to lipid retention in hypoxic trophoblasts, highlighting a key role for CGI58 in placental lipid mobilization. Additional detailed experiments might be warranted to confirm these results in conditions that mimic hypoxic placental injury in human diseases.

We were intrigued by the enhanced retention of LCPUFA in CGI58-KO placentas, which was reversed by the placenta-specific overexpression of CGI58. This shift in retention of LCPUFA and very-long-chain PUFA was also seen in LDs of PNPLA2-KO mouse hepatocytes, particularly after fasting (82). It has been shown that PNPLA2 prefers hydrolysis at the sn-2 position, resulting in sn-1,3 DAG (41). This specificity is broadened to the sn-1 position in the presence of CGI58. Further, PNPLA2 favors hydrolysis of long-chain fatty acids, possibly preferring hydrolysis of esters with mono- or polyunsaturated fatty acids (41). Together, these observations may explain our observation of the “right shift” in retained fatty acids in CGI58-KO mice. Our complete understanding of PNPLA2 and CGI58 function in TAG hydrolysis and the potential preference of LCPUFA await a high-resolution analysis of CGI58/PNPLA2 protein complexes on the LD surface. Regardless, the retention of placental LCPUFA is consistent with the increased retention of LCPUFA and decreased fetal levels of the LCFUFAs AA and DHA in the growth-restricted fetus (83, 84).

While our work served to identify key regulators of lipolysis in trophoblastic LDs, our use of a global CGI58 KO prohibited us from assessing the fetal consequences of CGI58 deficiency in the placenta. Indeed, CGI58-KO mice exhibited lipid accumulation in fetal organs, such as hepatomegaly and hepatosteatosis (48, 74), which might have impeded assessment of overall fetal growth. Such functional assessment awaits the generation of placenta-specific CGI58 KO. Further, we focused on the interaction between PNPLA lipases and their cofactor CGI58, yet other known regulators of lipolysis at the LD, including other PNPLA members, may be relevant to efflux of neutral lipids from trophoblasts and the reduced efflux in hypoxia (85, 86). HSL regulates the second step in TAG hydrolysis, converting DAG to MAG, a process independent of CGI58 activity (57, 87). While the level of HSL was below the level of detection in our assays, others have reported the expression of HSL in the placenta (88–90). Other proteins have been implicated in attenuation of PNPLA2 hydrolysis, including hypoxia-inducible gene-2, also known as hypoxia-inducible LD-associated protein, and its ortholog G0S2, which directly binds PNPLA2 through the patatin domain-containing region of PNPLA2 and inhibits its TAG hydrolase function (40, 91–96). Other potential regulators of CGI58, including some of those identified through our protein-protein interaction screen, might play a role in trophoblastic lipid mobilization. Further, the coordinated action of these factors and cofactors might be perturbed in pregnancy complications that emanate from placental dysfunction. Similarly, the regulation of placental PNPLA2, PNPLA9, and CGI58 by insulin, as shown in other systems, remains to be clarified (97). Other relevant regulators, such as PPAR agonists, glucocorticoids, mTOR complex activators, or sirtuin 1, may also play a role in regulation of PNPLA2, PNPLA9, and CGI58 actions (36). Interestingly, PNPLA2 was recently shown to exhibit transacylase activity, critical for the formation of the antiinflammatory and antidiabetic palmitic acid esters of hydroxy stearic acids, and this activity was augmented (1.5-fold) by CGI58 (98). The relevance of these activities to placental LD balance remains to be elucidated.

## Methods

### Human placentas.

All participants provided informed consent to the use of deidentified and discarded term placentas (37–41 weeks) after uncomplicated labor and delivery, under protocols approved by the Institutional Review Board at the University of Pittsburgh. This included the use of whole placentas for trophoblast cell dispersal (protocol STUDY20050066) or for placental biopsies (protocol STUDY19120076). These biopsies (5 mm^3^) were obtained immediately after delivery, using a region of the placenta that is midway between the cord insertion and the placental margin and between the chorionic and basal plates, as we previously detailed (99). All tissues were either snap-frozen in liquid nitrogen and stored at −80°C until processed or fixed with 4% paraformaldehyde (Thermo Fisher Scientific) in 0.1 M phosphate-buffered saline (PBS) and embedded in paraffin or in cryomold. Sections (5 μm) were used for hematoxylin/eosin staining and microscopic analysis.

### Cell culture, ligands, and medium human chorionic gonadotropin and lactate dehydrogenase measurement.

Term placental cells were dispersed, and PHT cells were isolated using a modification of previously published trypsin-DNase-dispase/Percoll protocols, as previously described (100, 101). PHT cells were cultured at a density of 350,000 cells/cm^2^ in DMEM (Sigma-Aldrich) supplemented with 10% fetal bovine serum (Sigma-Aldrich) and 1% antibiotics, as we previously detailed (102). The BeWo trophoblast cells (ATCC, CCL-98) were maintained in F12K Kaighn’s modified medium (Gibco) supplemented with 10% bovine growth serum (HyClone) and antibiotics. The HEK293T cell line (ATCC CRL-11268) was used for lentivirus transductions, as we previously described (102) (and see below). Some of the cells were cultured on coverslips in less than 1% O_2_ hypoxic atmosphere, using a hypoxia chamber (Thermo Electron), as we have described (66, 103). Where indicated, after 24 hours in culture, the cells were supplemented for 24 to 48 hours by a fatty acid mix of LA (stock 200 mM) and OA (stock 100 mM, Sigma-Aldrich or Cayman Chemical) or various other fatty acids (stock 100 mM, all from Cayman Chemical), as indicated, including palmitic acid, AA, docosatetraenoic acid (adrenic acid), docopentaenoic acid, or DHA. All fatty acids were preincubated with serum-free medium containing 0.5% fatty acid-free BSA (Sigma-Aldrich) for 20 minutes at 37°C to allow the formation of a fatty acid–albumin complex and used at a final concentration of 100 μM in 0.1% ethanol. Culture medium levels of human chorionic gonadotropin (hCG) and lactate dehydrogenase were measured using enzyme immunoassay, as we previously described (102).

### Real-time PCR and quantitative PCR.

Total RNA from placentas, cells, or human tissues (FirstChoice Survey Panel catalog 6000, Ambion) was extracted using TRI reagent (Thermo Fisher Scientific) according to the manufacturer’s protocol. RNA samples were further purified with on-column RNase-free DNase (QIAGEN). Real-time quantitative PCR (RT-qPCR) was performed as we previously described, in duplicate, with SYBR Select Master Mix (Thermo Fisher Scientific, catalog 4472908), according to standard procedures, as we previously described, using the ViiA 7 Real-Time PCR System (Thermo Fisher Scientific) (104, 105). The results were calculated using the 2^−ΔCT^ method (106) and normalized to the expression of the housekeeping gene GAPDH or 3-monooxygenase/tryptophan 5-monooxygenase activation protein, zeta polypeptide (YWHAZ). The specificity of amplification was confirmed using a dissociation curve of each PCR product. Control H_2_O samples were included in each qPCR experiment. All primer sequences are provided in Supplemental Table 3.

For regular PCR, RNA was extracted as above and processed with DNase I (Ambion). For reverse transcription, total RNA (1 μg) was added to 4 μL High-Capacity RNA-to-cDNA Master Mix (Applied Biosystems) according to the manufacturer’s instructions and diluted into 100 μL. For PCR, we mixed cDNA (2 μL) with a dNTP mixture (2.5 mM) and forward and reverse primers as noted in Supplemental Table 3, along with 2 μL of Ex Taq buffer and 0.1 μL of Ex Taq HS (Takara Bio) in a 20 μL reaction volume. PCR was performed using 30 to 35 cycles of 10 seconds at 98°C, 30 seconds at 55°C, 1 minute at 72°C. The PCR product (8 μL) was loaded and electrophoresed in 2% DNA gel and visualized using a UVP BioImaging System (Ultraviolet Products) or ChemiDoc MP Gel Imaging System (Bio-Rad).

### Tissue immunofluorescence and laser-capture microdissection.

Immunofluorescence of PNPLA9 in human or mouse placental sections was performed as previously described (102). A similar procedure was used for detection of CGI58, including blocking in donkey serum for 2 hours and incubation with primary anti-CGI58 rabbit monoclonal antibody and a secondary antibody, as detailed in Supplemental Table 4. For laser-capture microdissection, frozen sections (7 μm) of cryomold-embedded mouse placentas were transferred to polyethylene naphthalate membrane slides (Thermo Fisher Scientific) and stained with toluidine blue. The Leica LMD7000 laser-capture microdissection system was used to visualize, circumscribe, and collect 3 tissue types: labyrinth, junctional zone, and decidua. RNA was extracted from the microdissections, using the RNeasy FFPE Kit (QIAGEN, catalog 73504) according to the manufacturer’s instructions. The SuperScript VILO synthesis kit enzyme (Thermo Fisher Scientific, catalog 11755500) was used for the reverse transcription reaction, according to the manufacturer’s instructions. qPCR was performed in duplicate as detailed above.

### Western immunoblotting.

The source of all antibodies is provided in Supplemental Table 4. Cultured cells were washed with cold PBS, scraped, pelleted, and lysed in PBS containing 0.25 M sucrose and 0.05% NP-40 or in a 50 mmol/L Tris-HCl buffer containing 150 mmol/L NaCl, 1% NP-40, 0.5% sodium deoxycholate, and 0.1% sodium dodecyl sulfate (pH 7.5). Both lysis buffers contained protease inhibitor (Thermo Fisher Scientific, catalog 1861278). Following lysis, the mix was centrifuged at 12,000*g* for 15 minutes at 4°C and the supernatant was collected. Snap-frozen mouse placentas were thawed and homogenized or sonicated in the lysis buffer as above. Total protein concentration was measured using the Pierce BCA Protein Assay Kit (Thermo Fisher Scientific). Protein aliquots (20–30 μg) were separated by 8% to 10% SDS-PAGE and transferred to PVDF membranes (Bio-Rad). Blots were blocked with 5% nonfat milk in Tris-buffered saline with 0.1% Tween 20 (TBST) for 0.5 to 1 hour and incubated with the relevant primary antibody overnight at 4°C. Following TBST washes, membranes were incubated with the appropriate secondary antibody (Supplemental Table 4) for 1 hour at room temperature and enhanced using WesternBright Sirius HRP substrate (Advansta). Chemiluminescence signal was detected using the ChemiDoc MP Gel Imaging System.

### Expression, purification, and cross-linking of CGI58 with Sulfo-SBED.

Human CGI58 expression sequence was PCR-amplified with primers designed to introduce BamHI and NotI sites using human CGI58 expression plasmid as a template (GE Healthcare Life Sciences, MHS1010-74037 clone 3920855). The PCR product was cloned into the pET-Duet1 plasmid (Addgene plasmid 71146) for bacterial expression. Mouse *Cgi58* was PCR-amplified with primers including a His tag and flanked by BamHI/XhoI sites for cloning into pcDNA3.1. Mouse *Pnpla2* expression sequence was PCR-amplified with primers that include NotI/KpnI sites for cloning into pFlag-CMV2 (Sigma-Aldrich). Mouse *Pnpla9* expression sequence was PCR-amplified with primers that include EcoRI/KpnI sites using mouse Pnpla9 expression plasmid as a template (GE Healthcare Life Sciences, MMM1013-202769194 clone 5329147). The PCR product was cloned into pFlag-CMV2. All constructs were verified by sequencing (GENEWIZ).

The plasmid pETDuet-1-His-CGI58 was transformed into Rosetta DE3 competent cells (Sigma-Aldrich), induced with 0.5 mM IPTG, and then grown for an additional 3 hours at 25°C. The cells were pelleted at 4,000*g* at room temperature and resuspended in PBS that contained 0.5% Triton X-100, 50 μg/mL lysozyme, and protease inhibitors (cOmplete ULTRA Tablets, Roche catalog 05892970001, Sigma-Aldrich). The mixture was sonicated and centrifuged at 100,000*g* for 1 hour at 4°C, the cell lysate was diluted to reduce the Triton X-100 concentration to 0.1%, and imidazole (5 mM) was added to the lysate. A tricorn column (Sigma-Aldrich) was packed with His-select beads (Sigma-Aldrich) and loaded with the lysate using an Äktaxpress system (GE Healthcare). CGI58 was then eluted with 300 mM imidazole after extensive washes with increasing concentrations of salt (20–80 mM).

Sulfo-SBED was used according to the manufacturer’s instructions (Thermo Fisher Scientific, catalog 33034). Briefly, Sulfo-SBED was dissolved in DMSO and added to purified CGI58 in a 5 M excess, then incubated at room temperature for 30 minutes, protected from light. The samples were desalted using 2 mL Zeba spin desalting columns (Thermo Fisher Scientific, catalog 89890), The protein was eluted and conjugation with Sulfo-SBED was confirmed by Western blot using streptavidin-HRP (Thermo Fisher Scientific, catalog 21130) and by quantifying biotin (Thermo Fisher Scientific, catalog 28005).

### Co-precipitation of CGI58-interacting proteins.

As a source of CGI58-interacting proteins, we homogenized WT C57BL/6 mouse placentas in a PBS buffer that contained 0.25 M sucrose, 0.05% NP-40 (Roche catalog 11332473001, Sigma-Aldrich), and protease inhibitors, as noted above. The homogenized tissue was incubated for 30 minutes on a rotating platform at 4°C and then centrifuged at 13,000*g* for 15 minutes. Labeled and cross-linked CGI58 protein was incubated with the placenta lysate overnight on a rocker at 4°C, protected from light. The mixture was transferred to a plastic well on ice and irradiated using a long-wave UV lamp (365 nm) for 10 minutes. The sample was transferred to a microcentrifuge tube, and 150 μL of Dynabeads MyOne Streptavidin T1 (Thermo Fisher Scientific, catalog 65601) was added and allowed to mix for 2 hours at 4°C. The beads were washed with a high-salt, 300 mM NaCl buffer and then with the homogenization buffer. The Dynabeads were resuspended in 1× SDS sample buffer and boiled at 95°C for 10 minutes before resolving on SDS-PAGE gel. Gel slices were cut and submitted to the University of Pittsburgh’s Biomedical Mass Spectrometry Center for processing and analysis. All proteins shown in Supplemental Table 2 were identified in the 65–80 kDa gel slice. A similar process was repeated for validating the interaction of CGI58 with PNPLA9, using 8% SDS-PAGE gel, followed by transfer to a PVDF membrane and probing for CGI58 and PNPLA9, using antibodies as noted earlier (Supplemental Table 4).

For co-precipitation of *purified* His-CGI58 and endogenous PNPLA9, we repeated the same procedure described above, using 2 WT C57BL/6 mouse placentas. After incubation, the lysate was precleared with Protein G Dynabeads (Thermo Fisher Scientific, catalog 10003) for 30 minutes at 4°C. The precleared lysate was mixed with 12 μg of purified CGI58 protein and 10 μg of anti-His antibody or mouse IgG as a negative control. The sample was mixed at 4°C overnight, and Protein G Dynabeads were then added for 2 hours at 4°C. The Dynabeads were washed and resuspended in 1× SDS sample buffer, then processed for Western immunoblotting with antibodies as above.

To assess the interaction of CGI58 with ectopically expressed tagged proteins, we used FuGENE 6 (Promega, catalog E2691) to transfect HEK293T cells with plasmids expressing mouse pcDNA3.1-CGI58, mouse pFlag-CMV2-PNPLA2, or mouse pFlag-CMV2-PNPLA9. Two days after transfection, the cells were collected and resuspended in a 100 mM NaCl buffer that contained 50 mM Tris-HCl pH 7.2, 1% Triton X-100, 2 mM β-mercaptoethanol, and protease inhibitors (as above). The samples were incubated on a rotating platform at 4°C and centrifuged at 13,000*g* for 15 minutes. The lysate was precleared with Protein G Dynabeads for 30 minutes on a mixer at 4°C. The precleared lysate was transferred to another tube, and 10 μg of anti-His or anti-FLAG antibody (Supplemental Table 4) was added per immunoprecipitation. The samples were mix-incubated at 4°C overnight. Protein G Dynabeads were mixed the next day for 2 hours at 4°C. The beads were washed, resuspended in SDS sample buffer, and processed for immunoblotting with 2 μg/mL anti-His antibody or 2 μg/mL anti-FLAG antibody (Supplemental Table 4).

### KD or CRISPR/Cas9-mediated protein KO in BeWo cells.

We have previously described the generation of stably expressing, doxycycline-inducible Cas9 in BeWo cells and their use to create PNPLA2-KO and PNPLA9-KO BeWo cells (102). The Cas9 plasmid (Addgene plasmid 50661) was generated by the Lander and Sabatini Labs (107). The plasmid pLKO5.sgRNA.EFS.GFP (Addgene plasmid 57822) was used for the PNPLA2 guide, and the plasmid pLKO5.sgRNA.EFS.tRFP657 (Addgene plasmid 57824) was used for the *Pnpla9* guide. Both plasmids were generated by the Ebert Lab (108).

For KD of CGI58 we used shRNA lentiviruses that were produced using our published protocol (109). Briefly, we transiently transfected HEK293T cells with a combination of 3 lentiviral plasmids, including pLV-CGI58 shRNA (RHS4430-200174486, sequence 5′-GCATTAAAGCAGCGTATC-3′, Horizon Discovery, Dharmacon) alongside pMD2.G (Addgene plasmid 12259) and psPAX2 (Addgene plasmid 12260). After 60 hours, the lentiviruses in the conditioned media were pelleted by centrifugation at 18,000*g* for 2 hours at 4°C and resuspended in 100 μL of PBS. BeWo cells were transduced with concentrated lentiviruses in the presence of 8 μg/mL polybrene (Sigma-Aldrich; TR-1003-G). At 48 hours after transduction, BeWo cells were selected with 5 μg/mL puromycin (Invivogen; ant-pr-1) for 7 days to ensure the nontransduced WT cells were eliminated.

### Mice and breeding.

All mouse-related procedures and experimental protocols were approved by the IACUC of Magee-Womens Research Institute and the University of Pittsburgh (IACUC 19075519) and conducted in accordance with United States Public Health Service policy, as defined in the *Guide for the Care and Use of Laboratory Animals* (National Academies Press, 2011). *Pnpla9-*mutant mice (129S6/BL/6) were obtained from Jackson Laboratory as we previously detailed (102). *Pnpla2-* and *Cgi58-*mutant mice (129SV/BL/6) were provided by Rudolf Zechner (University of Graz, Graz, Austria) and Erin Kershaw (University of Pittsburgh, Pittsburgh, Pennsylvania, USA) and previously published (48). All data were validated using mice that were bred more than 6 times into the C57BL/6 strain. Timed breeding experiments were performed by pairing males and females overnight. Generation of *Pnpla2*/*Pnpla9*-DKO placentas was accomplished by mating *Pnpla2^KO^* *Pnpla9^Het^* males with *Pnpla2^Het^ Pnpla9^Het^* females. The morning after mating was considered E0.5 after verifying the presence of a vaginal plug. All mice were kept under standard conditions of 12-hour light/12-hour dark cycle and fed a regular rodent chow diet and water ad libitum. Pregnancy was confirmed by a 10% weight gain at E13.5. Dams were euthanized by CO_2_ followed by cervical dislocation on E17.5. Fetuses and placentas were delivered transabdominally and weighed. One half of each placenta was fixed using 4% paraformaldehyde and the other half snap-frozen and stored at −80°C for Western blot, TG, or lipidomic analysis. Genomic DNA was isolated from fetal tails using the HotSHOT (hot sodium hydroxide and Tris) method, and genotyping was performed by standard PCR (Verity, Applied Biosystems). The list of all primers used for genotyping is provided in Supplemental Table 5.

For in vivo exposure to hypoxia, timed pregnant mice (E11.5–E12.5) were exposed to hypoxic conditions of 11% O_2_ for 5 to 6 days, using a hypoxia chamber designed specifically for mouse experiments (Coy Laboratory Products). Following removal from the hypoxia chamber, some of the mice were immediately sacrificed while others underwent a period of reoxygenation prior to sacrifice, as detailed in the Results section. After sacrifice, fetuses and placentas were delivered transabdominally for use in histological and biochemical analysis, as noted earlier.

### Lentiviral transduction of blastocysts for CGI58 “rescue.”

*Cgi58*-Het mice in C57BL/6 were crossbred for at least 6 generations with ICR mice, creating *Cgi58*-Het mice in the ICR (Jackson Lab) background that were used for blastocyst manipulations. Blastocyst-specific CGI58 overexpression was performed using the procedure we previously described in detail (70). Briefly, CGI58 overexpression was performed using the following 3 lentiviral DNA plasmids: expression plasmid FUGW (Addgene plasmid 14883) for overexpression of CGI58 (or no expression sequences, as control) driven by EF1a promoter, envelope plasmid pLTR-G (Addgene plasmid 17532), and packaging plasmid pCD/NL-BH*DDD (Addgene plasmid 17531). Lentiviruses were produced in HEK293T cells (70), and lentiviral titers were quantified using ELISA (Zeptometrix 0801111B). ICR females were injected with pregnant mare serum gonadotropin on day 1 and with hCG on day 3, when breeding took place. On day 7 of the experiment, or E3.5, blastocysts were flushed from the uterus, processed for lentiviral transduction, and transferred back to pseudo-pregnant females, now considered E2.5. Pregnancy was confirmed by 10% weight gain on E13.5, and delivery was at E18.5. Note that, on average, 18.2 (range 5–20) blastocysts were transferred, yielding a mean of 8.2 (range 1–16) live pups. Placental and fetal *Cgi58* genotype was determined using the primers listed in Supplemental Table 5, which also confirmed that 25% of the newborns were *Cgi58* KO.

### Assessment of neutral lipids in tissues or cells.

Oil Red O (Sigma-Aldrich) or LipidTox (Thermo Fisher Scientific) was used for staining of neutral lipids, as we previously described (66), for quantification of lipid accumulation in mouse placentas. Sections were counterstained with hematoxylin and mounted with aqueous mounting medium. Images were acquired with a 90i widefield microscope (Nikon), and quantification of lipid staining in the placental labyrinth was done using Nikon NIS-Elements software.

We used BODIPY, diluted to 10 μg/mL in PBS (Molecular Probes), to quantify neutral LDs in cells, which were cultured on coverslips coated with 0.01% poly-l-lysine (Sigma-Aldrich) in a 12-well plate, as we previously described (66). The cells were rinsed with cold PBS, fixed in 2% paraformaldehyde for 20 minutes, and washed with PBS. Nuclei were stained with DAPI (Sigma-Aldrich) as described (66). Images were acquired with a Nikon A1R confocal system, and quantification of LDs was done using Nikon NIS-Elements software or ImageJ software (NIH). The total number, area, and average size of LDs stained by BODIPY, along with the total number of nuclei for each field, were recorded.

For quantification of TGs in tissues or cells, whole mouse placentas were homogenized in a 5% NP-40 buffer in 2 mL/100 mg tissue or 500 μL/100 mg protein for cells. The samples were heated at 90°C for 2 minutes, then cooled to room temperature. The heating and cooling steps were repeated twice, and the samples were centrifuged at 8,000*g* for 15 minutes at room temperature. The clear lysate was used to measure TG content, using a colorimetric Triglyceride Quantification Kit (BioVision) according to the manufacturer’s instructions. Absorbance was determined at 570 nm using a VersaMax spectrometry microplate reader (Molecular Devices).

### Reverse-phase column separation of TG.

To analyze TG in cultured trophoblasts or in mouse placental tissue, total lipids were extracted by the Folch procedure (110) and analyzed by a Dionex Ultimate 3000 HPLC coupled online to a Q-Exactive hybrid quadrupole-orbitrap mass spectrometer (Thermo Fisher Scientific). TGs were separated on a reverse-phase column [Luna 3 μm C18 (2) 100A, 150 × 1.0 mm, Phenomenex]at a flow rate of 0.065 mL/min. The column was maintained at 35°C. The analysis was performed using gradient solvents (A and B) containing 0.1% NH_4_OH. Solvent A was methanol and solvent B was propanol. The column was eluted for 2 minutes from 0% B to 2% B (linear), from 3 to 6 minutes with a linear gradient from 2% solvent B to 3% solvent B, then isocratically from 3 to 18 minutes using 3% solvent B, 18 to 35 minutes with a linear gradient from 3% solvent B to 40% solvent B, 35 to 60 minutes using a linear gradient from 40% to 55% solvent B, then isocratically from 60 to 65 minutes at 55% solvent B, then from 65 to 80 minutes from 55% to 0% B (linear), followed by equilibration from 80 to 90 minutes at 0% B.

### MS and MS/MS analysis of TGs.

MS and MS/MS analysis of TGs were performed mostly on a Q-Exactive hybrid quadrupole-orbitrap mass spectrometer, and part of experiments were performed on the ion trap LXQ (Thermo Fisher Scientific). TG cations were formed through molecular ammonium adduction (TG + NH_4_). Positional analysis of acyl chains in TG species was performed after collision-induced dissociation fragmentation of TGs (111, 112). Analysis was performed in positive-ion mode at a resolution of 140,000 for the full MS scan and 17,500 for the MS2 scan in a data-dependent mode with an inclusion list for TGs. The scan range for MS analysis was *m/z* 300–1,200 with a maximum injection time of 128 ms using 1 microscan. A maximum injection time of 500 ms was used for MS2 (high-energy collisional dissociation) analysis with collision energy set to 24. An isolation window of 1.0 Da was set for the MS and MS2 scans. Capillary spray voltage was set at 4.5 kV, and capillary temperature was 320°C. Sheath gas was set to 8 arbitrary units and the S-lens Rf level was set to 60.

### Statistics.

The data were analyzed using 1-way ANOVA with Tukey’s post hoc method (for all pairwise comparisons) or with Dunnett’s post hoc test (for comparison with control). For 2-group comparisons, 2-tailed *t* test was used. Data are presented as means ± SD where relevant. *P* < 0.05 was determined to be significant. All analyses were performed using Prism, version 9.4.1 (GraphPad).

### Study approval.

All experiments involving mice were approved by the IACUC at the University of Pittsburgh (protocols IS00002512 and IS00015519). All experiments involving the use of human tissue were approved by the Institutional Review Board at the University of Pittsburgh. This included the use of whole placentas for trophoblast cell dispersal (exempt protocol STUDY20050066) or for placental biopsies (protocol STUDY19120076, with written informed consent received prior to participation).

## Author contributions

JGS, MM, SYO, VEK, and YS conceived the study and designed the experiments. JGS, MM, SYO, TM, JPG, IB, ES, and YO performed the experiments. VAT and YYT performed the lipidomics analysis and analyzed data. JGS, VEK, and YS discussed and interpreted all results. JGS, MM, SYO, and YS wrote the manuscript.

## Supplementary Material

Supplemental data

## Version 1 05/22/2023

Electronic publication
