# Supplementary material for: The lipase cofactor CGI58 controls placental lipolysis
Source: JCI Insight. 2023 May 22;8(10):e168717. doi: 10.1172/jci.insight.168717 (PMC10322693; doi:10.1172/jci.insight.168717)
Supplement: Supplemental data [file jciinsight-8-168717-s087.pdf]

SUPPLEMENTAL MATERIAL: The lipase cofactor CGI58 controls placental lipolysis

Jennifer Guerrero-Santoro, Mayumi Morizane, Soo-Young Oh, Takuya Mishima, Julie P. Goff, Ibrahim Bildirici, Elena Sadovsky, Yingshi Ouyang, Vladimir A. Tyurin, Yulia Y Tyurina, Valerian E. Kagan, Yoel Sadovsky

Contents: Supplemental Figures 1 – 5
Supplemental Tables 1 – 5

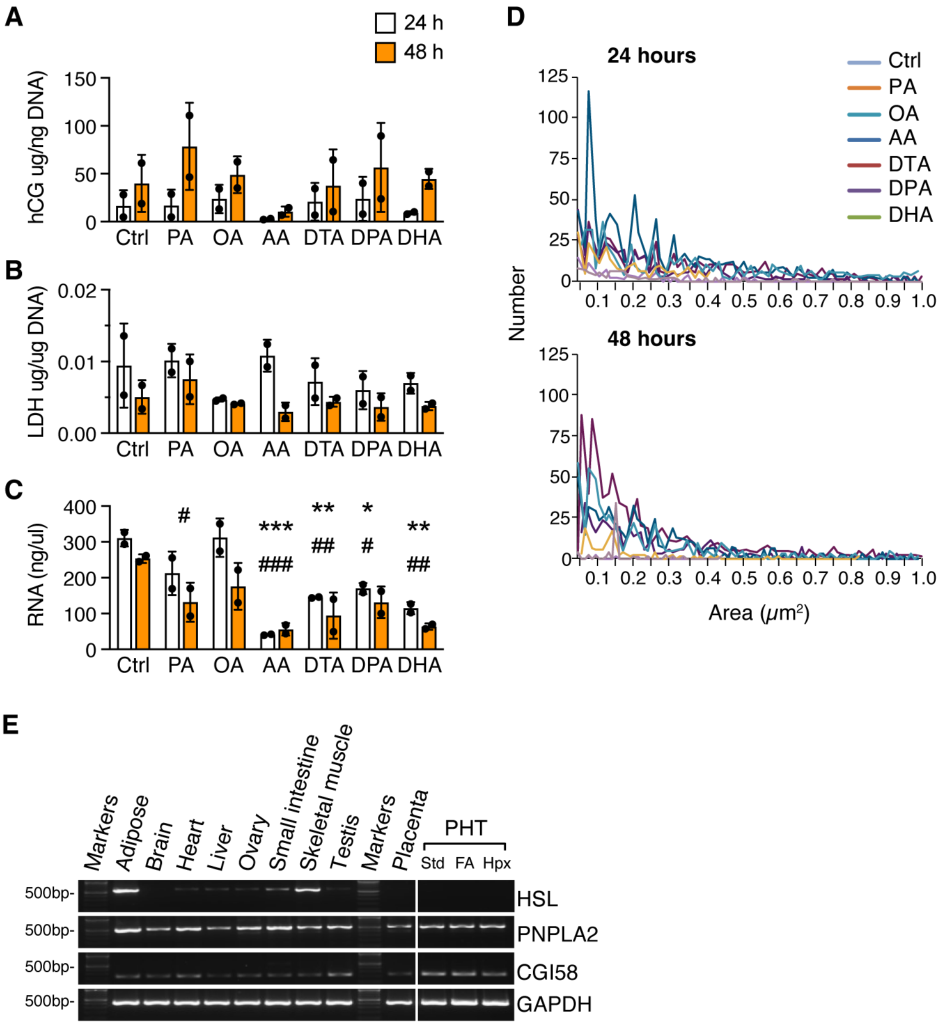

Supplemental Figure 1. The effect of fatty acids on LD accumulation in cultured PHT cells. PHT cells were cultured for 24-48 h in DMEM that contained added vehicle (ethanol) or added fatty acids (100 μM each) as noted in the figure with abbreviations as detailed in Methods. The effect of the fatty acids on (**A**) medium levels of hCG. (**B**) medium levels of LDH. (**C**) total RNA, and (**D**) LD area, was determined as detailed in Methods. n=2-3, ANOVA with *post hoc* Dunnett's test comparing each result to control at the same time point. In (**C**), * denotes differences at 24 h, and # denotes differences at 48 h. (**E**) The expression of mRNA for PNPLA2, HSL, and CGI58 in tissues or PHT cells cultured in standard conditions, in the presence of 200 μM LA/OA 2:1 mixture, or in hypoxia (Hpx, O2<1%). n=3.

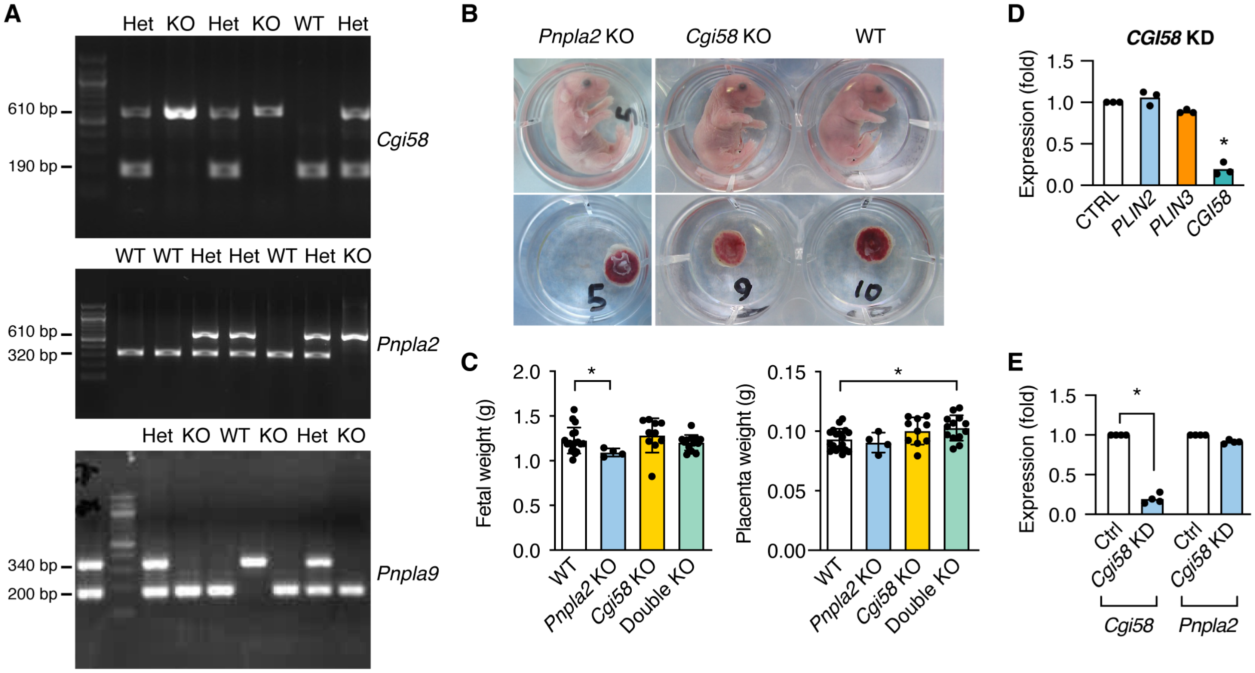

Supplemental Figure 2. The effect of deletion of Pnpla2 or Cgi58 or both on mouse placentas and human PHT cells. (**A**) PCR-based genotypic analysis of mouse lines used in the study. (**B**) Images of mouse fetuses and placentas (E17.5). Note the lighter color of the *Cgi58* KO placenta. (**C**) The distribution of mouse fetal and placental weights, by genotype. n=4-14, * $p<0.05$, ANOVA with *post hoc* Tukey's test. (**D**) The effect of *CGI58* KD on PLIN2/3 RNA in PHT cells. n=3, * $p<0.01$, ANOVA with *post hoc* Dunnett's test. (**E**) The effect of *CGI58* KD on PNPLA2 mRNA in PHT cells. n=4, * $p<0.001$, t-test.

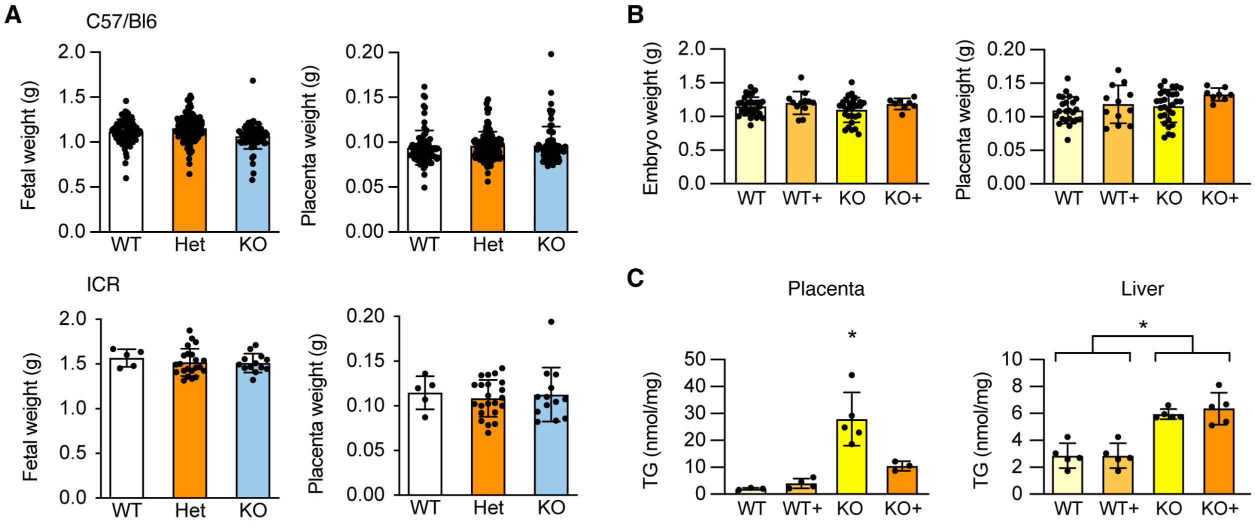

Supplemental Figure 3. Placenta-selective overexpression of CGI58 rescues the lipid accumulation phenotype in Cgi58 KO mice. (**A**) Fetal and placental weights in C57Bl/6 fetuses (upper panel) *vs* ICR fetuses (lower panel), analyzed by genotype (n=5-21). (**B**) Fetal and placental weights of WT or *Cgi58* KO fetuses and placentas, with or without overexpression of CGI58 (n=5-31). (**C**) The level of TGs (expressed as nmol/mg) in the placenta or fetal liver from WT or Cgi58 KO mice with or without placenta-specific overexpression of CGI58. (Placenta n=3-5, Fetal Liver n=5, * p<0.01, ANOVA with *post hoc* Tukey's test).

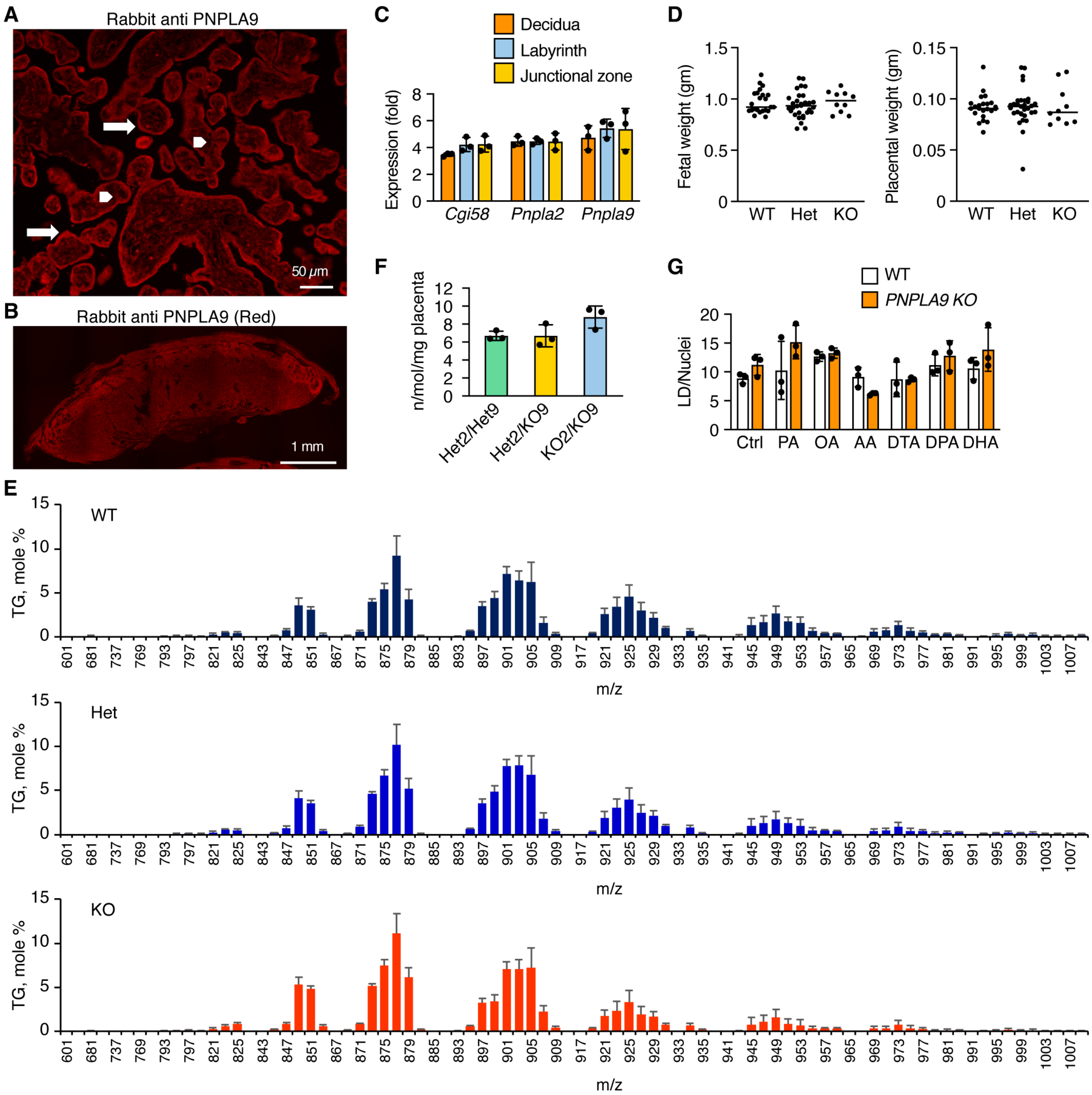

Supplemental Figure 4. The expression and function of Pnpla9 in placental lipid accumulation. (**A**) The expression of PNPLA9 in human term placental villi. The white arrows denote lipid signal in villous trophoblasts, and the white arrowhead denotes lipids in the villous core. (**B**) The expression of PNPLA9 in the mouse placenta. (**C**) The expression of *Cgi58*, *Pnpla2*, and *Pnpla9* in the mouse placental layers, analyzed using laser capture microscopy (n=3, p=ns, ANOVA with *post hoc* Tukey's test). (**D**) Fetal and placental weight by *Pnpla9* genotype (n=10-32, p=ns, ANOVA). (**E**) TG levels (expressed by m/z ratio) in *Pnpla2*/*Pnpla9* DKO mice (n=3, p=ns, ANOVA with *post hoc* Tukey's test). (**F**) MS/MS analysis of TAG's fatty acid distribution across *Pnpla9* mouse genotypes, performed as detailed in Methods. (**G**) Accumulation of LDs, normalized to the number of nuclei, in *PNPLA9* KO BeWo human trophoblast line in the absence or presence of added fatty acids for 24 h, analyzed as described in Methods (n=3, p=NS, t test).

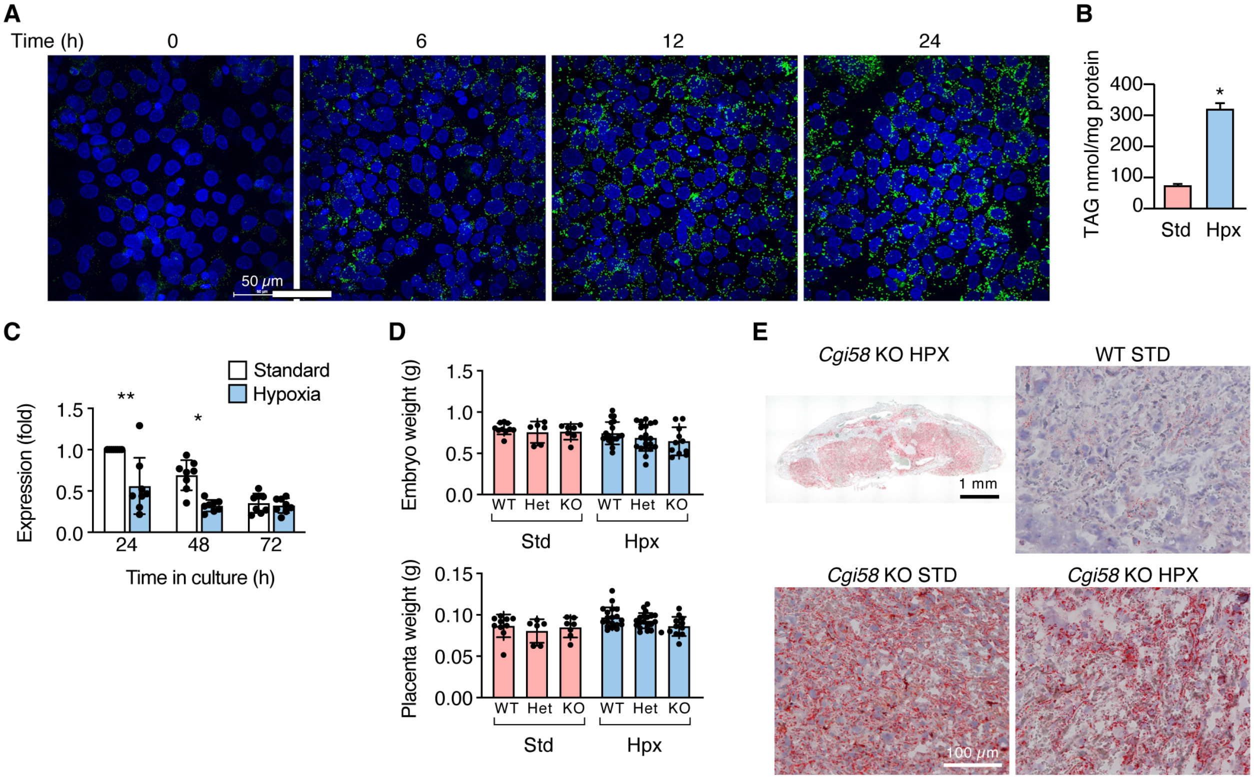

Supplemental Figure 5. The function of CGI58 and PNPLA9 in the hypoxic placenta. (**A**) The effect of hypoxia on LD accumulation in hypoxic (0-24 h) PHT cells. (**B**) The level of TAG in PHT cells after 24 h of hypoxia (n=3). (**C**) The expression of *CGI58* mRNA during hypoxia in trophoblasts, determined using RT-qPCR (n=5-6, * $p<0.05$, ** $p<0.01$, ANOVA with *post hoc* Tukey's test). (**D**) The distribution of mouse fetal and placental weights, by *Cgi58* genotypes (n=6-14, p=ns, ANOVA with *post hoc* Tukey's test). (**E**) The accumulation of LDs in the E17.5 mouse placental labyrinth, exposed to standard or hypoxic (O_2=11%) conditions between E12.5-E17.5, analysis by *Cgi58* genotype.

Supplemental Table 1. MS/MS analysis of TAG fatty acid composition in *Cgi58* WT vs KO placentas.

Fatty acid composition	m/z	WT (n=10) pmol/mg protein	CGI58 KO (n=10) pmol/mg protein	p-value
C20:4/C18:2/C16:1	894	219 ± 118	683 ± 220	<0.001
C20:4/C18:2/C16:0	896	1164 ± 747	1425 ± 1724	<0.05
C22:6/C16:0/C16:0	896	386 ± 333	635 ± 275	ns
C20:4/C18:1/C16:0	898	586 ± 180	949 ± 315	ns
C20:4/C18:0/C16:0	900	1206 ± 649	1815 ± 635	<0.05
C18:2/C18:1/C18:0	902	720 ± 731	1294 ± 1468	ns
C20:4/C20:4/C16:0	920	960 ± 279	3729 ± 1244	<0.001
C22:6/C18:1/C16:0	922	863 ± 253	1597 ± 687	<0.001
C22:6/C18:0/C16:0	924	897 ± 429	2489 ± 1126	<0.001
C22:5/C18:0/C16:0	926	543 ± 370	1583 ± 1151	<0.05
C22:6/C20:4/C16:0	944	478 ± 228	2819 ± 1611	<0.001
C22:6/C18:2/C18:1	946	720 ± 220	2764 ± 808	<0.001
C22:6/C18:0/C18:0	948	588 ± 208	2888 ± 1015	<0.001
C22:6/C18:1/C18:0	950	277 ± 247	1653 ± 1396	<0.01
C22:6/C20:4/C18:2	968	355 ± 136	4540 ± 1091	<0.001
C22:6/C22:6/C18:2	968	257 ± 167	907 ± 588	<0.01
C22:6/C20:4/C18:1	970	283 ± 119	1448 ± 516	<0.001
C22:6/C20:4/C18:0	972	196 ± 26	896 ± 336	<0.001
C22:6/C20:3/C18:0	974	202 ± 271	621 ± 477	<0.05
C22:6/C22:6/C18:2	992	176 ± 89	3608 ± 1122	<0.001
C22:6/C22:6/C18:1	994	107 ± 55	1186 ± 521	<0.001
C18:0/C22:6/C22:6	996	90 ± 63	1041 ± 508	<0.001
C18:0/C22:5/C22:6	998	109 ± 186	592 ± 495	<0.01

Supplemental Table 2. CGI58-interacting proteins (65-80 kDa) in mouse placental lysate

Protein symbol	Description
2AAA	Serine/threonine-protein phosphatase 2A 65 kDa regulatory subunit A alpha isoform
A1BG	Alpha-1B-glycoprotein
A2AAN2	Signal recognition particle subunit SRP68
A2BFF5	Cytoplasmic dynein 1 intermediate chain 2
ACADV	Very long-chain specific acyl-CoA dehydrogenase, mitochondrial
ACOC	Cytoplasmic aconitate hydratase
ACSL4	Long-chain-fatty-acid--CoA ligase 4
ACTB	Actin, cytoplasmic 1
ACTN4	Alpha-actinin-4
AMOT	Angiomotin
ANM5	Protein arginine N-methyltransferase 5
AP3B1	AP-3 complex subunit beta-1
ARHG1	Isoform 5 of Rho guanine nucleotide exchange factor 1
ATG7	Ubiquitin-like modifier-activating enzyme
B8JK32	Heterogeneous nuclear ribonucleoprotein M
CAN2	Calpain-2 catalytic subunit
CAND1	Cullin-associated NEDD8-dissociated protein 1
COR1C	Coronin-1C
CUL1	Cullin-1
CUL3	Cullin-3
CUL5	Cullin-5
D3Z4E3	DEAH (Asp-Glu-Ala-His) box polypeptide 32, isoform CRA_b
DDX1	DDX1_MOUSE ATP-dependent RNA helicase D
DESP	Desmoplakin
DHX15	Putative pre-mRNA-splicing factor ATP-dependent RNA helicase DHX15
DP13A	DCC-interacting protein 13-alpha
DPYL2	Dihydropyrimidinase-related protein 2
E9PVA6	ARF GTPase-activating protein GIT2
EF1A1	Elongation factor 1-alpha 1
EHD4	EH domain-containing protein 4
ERF3A	Eukaryotic peptide chain release factor GTP-binding subunit
ERG7	Lanosterol synthase
EX3L4	Exocyst complex component 3-like protein 4
F8WJE0	Deoxynucleoside triphosphate triphosphohydrolase SAMHD1
FBXW8	F-box/WD repeat-containing protein 8
FLNB	Filamin-B
G3X8X7	Vacuolar protein sorting 16 (Yeast)
G3X8Y7	Long-chain fatty acid transport protein 3
G3X957	Liprin-beta-2

Protein symbol	Description
G3XA10	Heterogeneous nuclear ribonucleoprotein U
GCYB1	Guanylate cyclase soluble subunit beta-1
GELS	Isoform 2 of Gelsolin
GNPAT	Dihydroxyacetone phosphate acyltransferase
GTPB1	GTP-binding protein 1
GUAA	GMP synthase [glutamine-hydrolyzing]
H7BX01	Dynamin-like 120 kDa protein, mitochondrial
HOOK3	Protein Hook homolog 3
IPO5	Importin-5
IQGA1	Ras GTPase-activating-like protein
K1C12	Keratin, type I cytoskeletal 12
K1C16	Keratin, type I cytoskeletal 16
K1C42	Keratin, type I cytoskeletal 42
K22E	Keratin, type II cytoskeletal 2 epidermal
K2C1	Keratin, type II cytoskeletal 1
K2C1B	Keratin, type II cytoskeletal 1b
K2C4	Keratin, type II cytoskeletal 4
K2C7	Keratin, type II cytoskeletal 7
K2C75	Keratin, type II cytoskeletal 75
K2C8	Keratin, type II cytoskeletal 8
KPCI	Protein kinase C iota type
LPP	Lipoma-preferred partner homolog
LYRIC	Protein LYRIC
MYO1C	Unconventional myosin-Ic
NIBL1	Niban-like protein 1
NUB1	NEDD8 ultimate buster 1
O88325	Alpha-N-acetylglucosaminidase
PDC6I	Programmed cell death 6-interacting protein
PDE10	cAMP and cAMP-inhibited cGMP 3',5'-cyclic phosphodiesterase 10A
PDLI5	PDZ and LIM domain protein 5
PEG10	Retrotransposon-derived protein
PLAK	Junction plakoglobin
PLAP	Phospholipase A-2-activating protein
PLPL9	85/88 kDa calcium-independent phospholipase (Pla2g6)
PTN11	Isoform 2 of Tyrosine-protein phosphatase non-receptor type 11
Q3U741	DEAD (Asp-Glu-Ala-Asp) box polypeptide 17, isoform CRA_a
Q3UW64	Bifunctional UDP-N-acetylglucosamine 2-epimerase/N-acetylmannosamine kinase
Q6NZD2	Sorting nexin 1
Q7TNL7	Dual specificity protein phosphatase
Q8BML9	Glutaminyl-tRNA synthetetase

Protein symbol	Description
Q8C605	6-phosphofructokinase
RAGP1	Ran GTPase-activating protein 1
RIR1	Ribonucleoside-diphosphate reductase large subunit
RUFY3	Isoform 2 of Protein RUFY3
S4R165	Ankyrin-3
SPHK2	Sphingosine kinase 2
STAT1	Signal transducer and activator of transcription 1
STAT3	Signal transducer and activator of transcription 3
SYEP	Bifunctional glutamate/proline--tRNA ligase
TBB3	Tubulin beta-3 chain
TBB6	Tubulin beta-6 chain
TNPO1	Transportin-1
TRI25	E3 ubiquitin/ISG15 ligase TRIM25
UBA6	Ubiquitin-like modifier-activating enzyme 6
UBP4	Ubiquitin carboxyl-terminal hydrolase 4

Supplemental Table 3. Primer sequences used for RT-PCR.

Gene/Direction	GeneBank accession no.	Site	Sequence (5' – 3')
Standard RT-PCR			
hPNPLA2 F	NM_020376	414-438	GAGGTATCTAAAGAGGCCCGGAAGC
hPNPLA2 R		947-927	AAAGCGCAGGCCATCCCGGTA
hCGI58 F	NM_016006	77-99	CATGGTGCCCTACGTCTATATCA
hCGI58 R		311-291	ACAGGTCTGTTGGTGCAAAGA
hHSL F	NM_005357.2	2000-2019	TTTGAGATGCCACTGACTGC
hHSL R		2127-2108	TCACTGTCCTGTCCTTCACG
Quantitative RT-PCR			
hPNPLA2 F	NM_020376	252-270	GCACCCCTCCTTCAACCTG
hPNPLA2 R		383-363	ATGACATTCTCGCCGTCTGAC
mPNPLA2 F	NM_01163689	213-232	CAACGCCACTCACATCTACG
mPNPLA2 R		310-290	CAATAATGTTGGCACCTGCTT
hCGI58 F	NM_016006	468-488	ATTCTTGGCTGCTGCTTACTC
hCGI58 R		597-575	TCTGATCCAAACTGGAATTGGTC
mCGI58 F	NM_026179	893-913	TGTTTGAAGATGACACGGTGA
mCGI58 R		1023-1004	ACCTATCCGCTGAAGCATTG
hPNPLA9 F	NM_003560	700-719	CGTCTTCCATTATGCTGTCC
hPNPLA9 R		798-779	AGCCCTTGGTTATTCACCTG
mPNPLA9 F	NM_016915	873-894	CAACAAAGGAGAGACTGCCTTC
mPNPLA9 R		988-969	TCAGCCCTTGGTTGTTTACC

Supplemental Table 4. List of antibodies used in this study.*

Protein	Species	Concentration/ Dilution	Vendor	Catalog	RRID
PNPLA2	Rabbit	Variable	Cell Signaling	2138	AB_2289810
PNPLA2	Rabbit	Variable	Cell Signaling	2439	AB_2167953
PNPLA9	Rabbit	1:50 (IF)	Sigma Aldrich	HPA001171	AB_1079145
PNPLA9	Mouse	1 μg/ml (WB)	Santa Cruz	sc-376563	AB_11150308
CGI58	Goat	Variable	Sigma	SAB2500231	AB_10695738
CGI58	Mouse	1:250 (IP)	Abnova	H00051099-1F3	AB_509070
CGI58	Mouse	1:1,000	Abgent	AT1012a	AB_1551136
Actin	Mouse	1:10,000 (WB)	Millipore	MAB1501	AB_2223041
Actin	Goat	1:1,000	Santa Cruz	sc1616	AB_630836
Anti-Rabbit (594)	Donkey	1:300 (IF)	Invitrogen	A21207	AB_141637
Anti-Mouse (HRP)	Goat	1:10,000 (WB)	Thermo-Fisher	ab6789	AB_955439
Anti-Mouse (HRP)	Goat	1:10,000 (WB)	Jackson Immun	115-035-146	AB_2307392
Anti-Goat-HRP	Donkey	1:10,000 (WB)	Jackson Immun	705035003	AB_2340390
FLAG	Mouse	2 μg/ml	Sigma	F3165	AB_259529
His	Mouse	2 μg/ml	GenScript	A00186-100	AB_914704

** Note that over the course of the project several antibodies might have been used.*

Supplemental Table 5. Primer sequences used for genotyping.

**Name/Direction**	**Species**	**Product length (bp)**	**Sequence (5' – 3')**
PNPLA9 F	Mouse	-	CAAATGCACGTTTGGTGTGT
PNPLA9 R1 WT	Mouse	340	CTCCCTGACCAGGACTGACT
PLA2G6 R2- KO	Mouse	200	CTCCAGACTGCCTTGGGAAAA
PNPLA2 F	Mouse	-	AGAGAGAGAAGCTGAAGCCTGG
PNPLA2 R WT	Mouse	320	GCCAGCGAATGAGATGTTCC
PNPLA2 -Neo (KO) R	Mouse	610	CTGCGTGCAATCCATCTTGT
CGI58 F WT	Mouse	-	GTCATGGTTGTGGGGAAA C
CGI58 R1 WT	Mouse	190	AGGAAGGGGTATTCTGCAGG
CGI58 R2 KO	Mouse	610	CTTCTTCCAGCTGCTTCTGC
